# Successful Extraction of Guidewire Entrapped in the Tricuspid Valve Leaflet Using a Laser Sheath

**DOI:** 10.1016/j.jaccas.2022.10.009

**Published:** 2022-11-08

**Authors:** Shogo Sakamoto, Moritoshi Irishio, Yoshihiro Nakatani, Toru Kataoka, Daiju Fukuda

**Affiliations:** a Department of Cardiovascular Medicine, Bellland General Hospital, Naka-ku, Sakai, Osaka, Japan; b Department of Cardiovascular Medicine, Osaka Metropolitan University Graduate School of Medicine, Abeno-ku, Osaka, Japan

**Keywords:** guidewire complication laser sheath, leadless pacemaker

## Abstract

Right ventriculography is a necessary step for implantation of leadless pacemaker and is considered a safe procedure. However, an inappropriate manipulation of the guidewire can lead to serious complications. We present a case where the guide-wire was entrapped in the tricuspid valve, and its successful extraction using a laser sheath. (**Level of Difficulty: Advanced.**)

## Central Illustration

**Figure:**
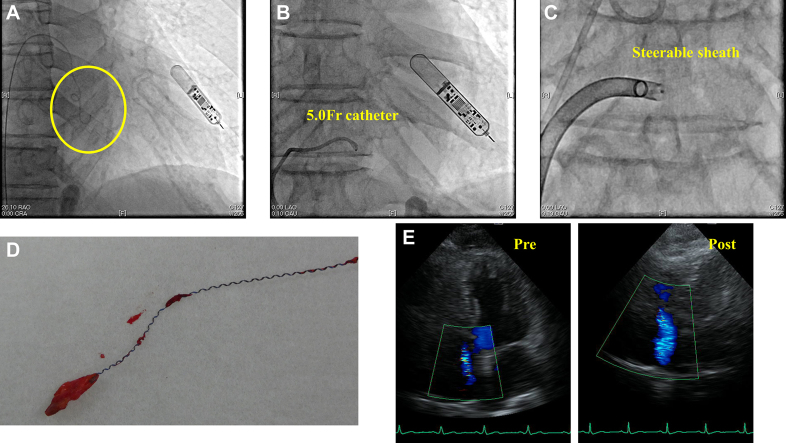

**Figure 1:**
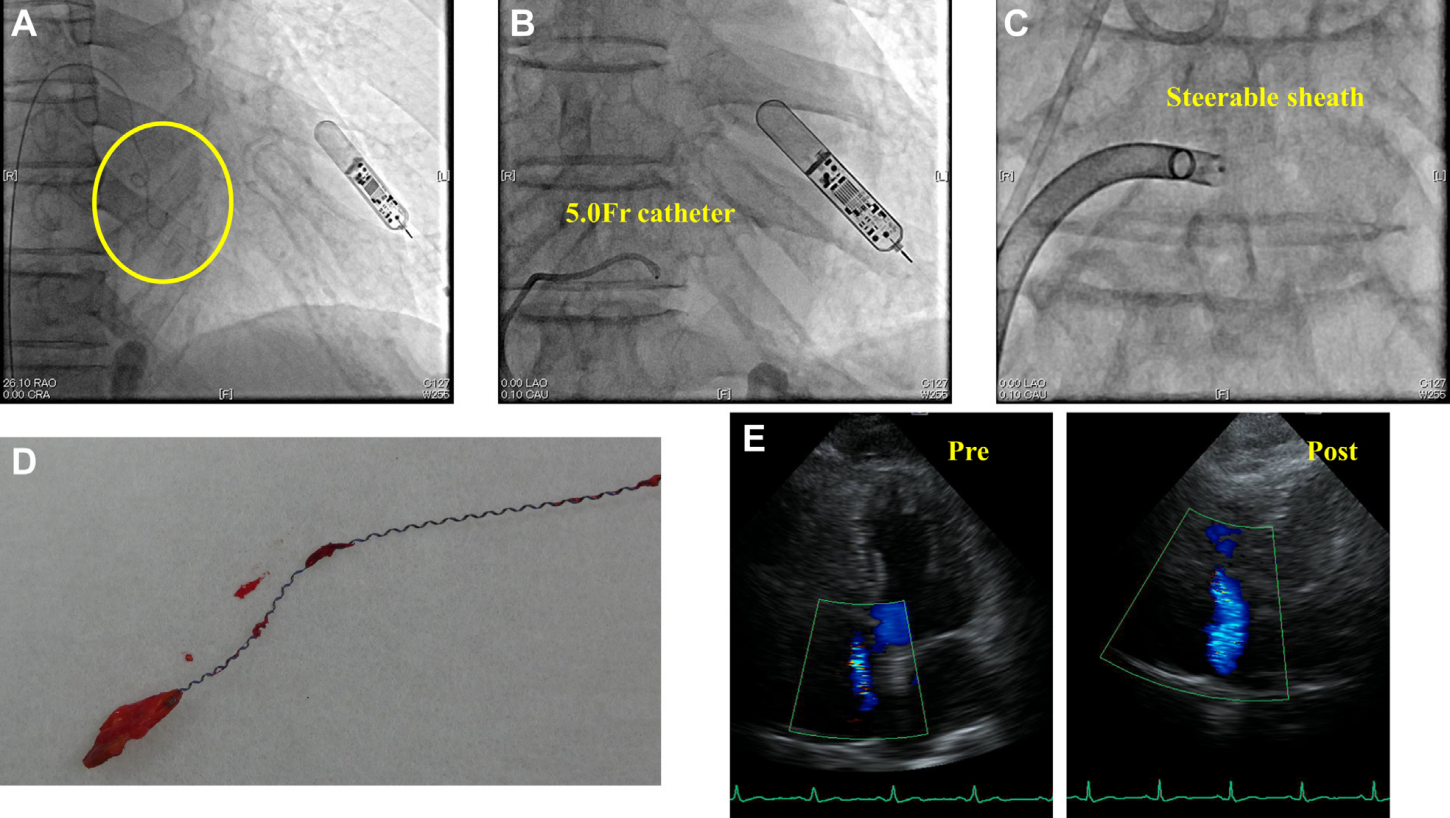
Fluoroscopy, the Extracted Guidewire, and Duplex Scanning **(A)** The guidewire was entrapped near the tricuspid valve **(yellow circle)**. **(B)** Attempting to rotate the guidewire using a 5.0-F catheter. **(C)** Adding counter traction to the guidewire using a steerable sheath. **(D)** The tricuspid valve attached to the surrounding coiled wire without an inner single filament core wire. **(E)** Comparison of the tricuspid regurgitation between preoperation and postoperation using duplex scanning.

A 76-year-old man with sick sinus syndrome underwent a leadless-pacemaker implantation. A 5.0-F pigtail catheter was passed over a 0.035-inch steel-tip J-tipped guidewire into the right ventricle. After performing a right ventriculography, we attempted to advance the catheter into the superior vena cava along a guidewire, but the guidewire was caught in the tricuspid valve leaflet during this procedure (Figure 1A). We first attempted to rotate and pull out the guidewire through a 5.0-F catheter, but the attempt failed (Figure 1B). We exchanged the introducer to a steerable sheath and attempted the counter-traction technique (Figure 1C), but all failed to release the guidewire. We were also unsure as to whether the guidewire was tangled at the Chiari network or at the ventricle near the tricuspid valve. Because the patient was under local anesthesia, we were unable to perform transesophageal echocardiography or prepare intracardiac echocardiography for usage at the time. Instead, we performed a right atriography and confirmed the guidewire lying at the ventricle near the septal cusp of the tricuspid valve, where it was stuck (Video 1). We considered pulling out the guidewire using force and added more traction using a snare catcher with the support of the steerable sheath, but it was unsuccessful (Video 2). Upon discussion with cardiac surgeons, open-heart surgery was considered high risk because of asbestosis. We decided to use a laser sheath (total length: 60 cm) for extraction, which has a slightly longer effective length than a mechanical sheath (46 cm). We inserted a 12-F laser sheath along the guidewire and delivered 300 pulses 3 times, and were successfully able to release the guidewire without any major surgical interventions (Video 3). A closer look at the retrieved guidewire revealed that the inner single filament wire had been fractured. Pathological examination of the tissue attached to the coil wire revealed it to be tricuspid valve tissue, along with right ventricular papillary muscle and chorda tendineae (Figure 1D). After confirming that there was no cardiac tamponade or atrioventricular block, we implanted the leadless pacemaker as planned.

The patient was discharged 8 days later without any complications. However, postimplantation duplex scanning revealed a newly generated tricuspid regurgitation that was not observed preimplantation (Figure 1E). This damage was likely caused by injuries to the tricuspid valve, papillary muscle, and chorda tendineae during the extraction.

A J-tipped guidewire is frequently used in various medical practices, and several complications related to the guidewire and its solutions are reported. When all solutions for extraction of stuck guidewire fails, surgical removal is usually the next choice.^1^ By using a laser sheath—a novel, minimally invasive approach—we were able to successfully remove the entrapped guidewire and avoid open surgery in a high-risk case. Nonetheless, the possibility of valve injury should be noted when using this method, and therefore, it should be considered as a bail-out strategy after consulting with cardiovascular surgeons.

## Funding Support and Author Disclosures

The authors have reported that they have no relationships relevant to the contents of this paper to disclose.

## Appendix

Supplemental Video 1 Right atriography revealed the guidewire caught at the ventricle near the septal tricuspid valve.

Supplemental Video 2 Attempted removal of the guidewire by adding traction using a snare catcher with the support of steerable sheath. In the process of pulling the guidewire, the soft tip of steerable sheath was deformed.

Supplemental Video 3 Successful removal of the guidewire using a laser sheath. The laser sheath irradiates the tip of guidewire with a laser, leading to an extraction of the guidewire from the tricuspid valve leaflet.
